# Supplementary material for: Association of Toll-like receptor 7 variants with life-threatening COVID-19 disease in males: findings from a nested case-control study
Source: eLife. 2021 Mar 2;10:e67569. doi: 10.7554/eLife.67569 (PMC7987337; doi:10.7554/eLife.67569)
Supplement: Reporting standard 1. [file elife-67569-repstand1.docx]

STROBE Statement—checklist of items that should be included in reports of observational studies

|  | **Item No.** | **Recommendation** | **Page  No.** | **Relevant text from manuscript** |
| --- | --- | --- | --- | --- |
| **Title and abstract** | 1 | (*a*) Indicate the study’s design with a commonly used term in the title or the abstract | 1-2 | Methods: This is a nested case-control study ….. |
|  |  | (*b*) Provide in the abstract an informative and balanced summary of what was done and what was found | 2 | See in the Abstract |
| **Introduction** | | | |  |
| Background/rationale | 2 | Explain the scientific background and rationale for the investigation being reported | 4 | Coronavirus disease 2019 (COVID-19), a potentially severe systemic disease caused by coronavirus SARS-CoV-2, is characterized by... |
| Objectives | 3 | State specific objectives, including any prespecified hypotheses | 5 | Here, we performed a nested case-control study within our prospectively recruited GEN-COVID cohort with the aim to... |
| **Methods** | | | |  |
| Study design | 4 | Present key elements of study design early in the paper | 5 | See Patients and samples  section |
| Setting | 5 | Describe the setting, locations, and relevant dates, including periods of recruitment, exposure, follow-up, and data collection | 5 | A subset of 156 young (<60 years) male COVID-19 patients was selected from... |
| Participants | 6 | (*a*) *Cohort study*—Give the eligibility criteria, and the sources and methods of selection of participants. Describe methods of follow-up  ***Case-control study***—Give the eligibility criteria, and the sources and methods of case ascertainment and control selection. Give the rationale for the choice of cases and controls  *Cross-sectional study*—Give the eligibility criteria, and the sources and methods of selection of participants | 5 | Cases were selected according... |
|  |  | (*b*) *Cohort study*—For matched studies, give matching criteria and number of exposed and unexposed  *Case-control study*—For matched studies, give matching criteria and the number of controls per case |  |  |
| Variables | 7 | Clearly define all outcomes, exposures, predictors, potential confounders, and effect modifiers. Give diagnostic criteria, if applicable | 5 | See Patients and samples  section |
| Data sources/ measurement | 8* | For each variable of interest, give sources of data and details of methods of assessment (measurement). Describe comparability of assessment methods if there is more than one group | 5 | See Patients and samples  section |
| Bias | 9 | Describe any efforts to address potential sources of bias | 6 | A similar cohort from the second wave, composed of 83 young male COVID-19 patients, was used to expand the cohort. |
| Study size | 10 | Explain how the study size was arrived at | 5 | A subset of 156 young (<60 years) male COVID-19 patients was selected from |

Continued on next page

| Quantitative variables | 11 | Explain how quantitative variables were handled in the analyses. If applicable, describe which groupings were chosen and why | 5 | See Patients and samples  section |
| --- | --- | --- | --- | --- |
| Statistical methods | 12 | (*a*) Describe all statistical methods, including those used to control for confounding | 6 | See Statistical Methods section |
|  |  | (*b*) Describe any methods used to examine subgroups and interactions | 6 | See Statistical Methods section |
|  |  | (*c*) Explain how missing data were addressed | 6 | See Statistical Methods section |
|  |  | (*d*) *Cohort study*—If applicable, explain how loss to follow-up was addressed  ***Case-control stu****dy*—If applicable, explain how matching of cases and controls was addressed  *Cross-sectional study*—If applicable, describe analytical methods taking account of sampling strategy | 6 | See Statistical Methods section |
|  |  | (*e*) Describe any sensitivity analyses | 6 | See Statistical Methods section |
| **Results** | | | | |
| Participants | 13* | (a) Report numbers of individuals at each stage of study—eg numbers potentially eligible, examined for eligibility, confirmed eligible, included in the study, completing follow-up, and analysed | 10 | By selecting for young (<60 year-old) males, we obtained... |
|  |  | (b) Give reasons for non-participation at each stage | 10 | By selecting for young (<60 year-old) males, we obtained... |
|  |  | (c) Consider use of a flow diagram | / | No |
| Descriptive data | 14* | (a) Give characteristics of study participants (eg demographic, clinical, social) and information on exposures and potential confounders | 9 | ...on the extreme phenotypic ends of the male subset of the Italian GEN-COVID cohort (https://sites.google.com/dbm.unisi.it/gen-covid). |
|  |  | (b) Indicate number of participants with missing data for each variable of interest | / | na |
|  |  | (c) *Cohort study*—Summarise follow-up time (eg, average and total amount) | / | na |
| Outcome data | 15* | *Cohort study*—Report numbers of outcome events or summary measures over time | */* | *na* |
|  |  | *Case-control study—*Report numbers in each exposure category, or summary measures of exposure | 10 | By selecting for young (<60 year-old) males, we obtained.. |
|  |  | *Cross-sectional study—*Report numbers of outcome events or summary measures | */* | *na* |
| Main results | 16 | (*a*) Give unadjusted estimates and, if applicable, confounder-adjusted estimates and their precision (eg, 95% confidence interval). Make clear which confounders were adjusted for and why they were included | / | na |
|  |  | (*b*) Report category boundaries when continuous variables were categorized | / | na |
|  |  | (*c*) If relevant, consider translating estimates of relative risk into absolute risk for a meaningful time period | / | na |

Continued on next page

| Other analyses | 17 | Report other analyses done—eg analyses of subgroups and interactions, and sensitivity analyses | / | na |
| --- | --- | --- | --- | --- |
| **Discussion** | | | | |
| Key results | 18 | Summarise key results with reference to study objectives | 10 | Toll-like receptor 7 (TLR7) was picked up as one of the most important susceptibility genes by LASSO Logistic Regression analysis |
| Limitations | 19 | Discuss limitations of the study, taking into account sources of potential bias or imprecision. Discuss both direction and magnitude of any potential bias | 12 | Since not all identified variants were functionally effective, the true percentage could be slightly lower in young males. |
| Interpretation | 20 | Give a cautious overall interpretation of results considering objectives, limitations, multiplicity of analyses, results from similar studies, and other relevant evidence | 12 | Overall, males with rare missense variants shown... |
| Generalisability | 21 | Discuss the generalisability (external validity) of the study results | 12 | The observation reported here may lead to consider TLR7 screening in severely affected male patients in order to start personalized interferon treatment for those with this specific genetic disorder. |
| **Other information** | |  | | |
| Funding | 22 | Give the source of funding and the role of the funders for the present study and, if applicable, for the original study on which the present article is based | 2 | See Funding Statement section |

*Give information separately for cases and controls in case-control studies and, if applicable, for exposed and unexposed groups in cohort and cross-sectional studies.

**Note:** An Explanation and Elaboration article discusses each checklist item and gives methodological background and published examples of transparent reporting. The STROBE checklist is best used in conjunction with this article (freely available on the Web sites of PLoS Medicine at http://www.plosmedicine.org/, Annals of Internal Medicine at http://www.annals.org/, and Epidemiology at http://www.epidem.com/). Information on the STROBE Initiative is available at www.strobe-statement.org.
